# MR imaging findings in some rare neurological complications of paediatric cancer

**DOI:** 10.1007/s13244-018-0628-z

**Published:** 2018-05-15

**Authors:** Tetsuhiko Okabe, Taiki Nozaki, Noriko Aida, Jay Starkey, Mikako Enokizono, Tetsu Niwa, Atsuhiko Handa, Yuji Numaguchi, Yasuyuki Kurihara

**Affiliations:** 1 Department of Radiology, St. Luke’s International Hospital, 9-1 Akashi-cho, Chuo-ku, Tokyo, 104-8560 Japan; 2 Department of Radiology, Yokohama City University, Kanagawa, Japan; 3 Department of Radiology, Kanagawa Children’s Medical Center, Kanagawa, Japan; 4 Department of Radiology, National Center of Neurology and Psychiatry, Tokyo, Japan; 5 Department of Radiology, Tokai University School of Medicine, Kanagawa, Japan; 6 MassGeneral Hospital for Children and Harvard Medical School, Boston, MA USA

**Keywords:** Paediatric cancer, Treatment, Neurological complication, MRI, imaging

## Abstract

**Abstract:**

Neurological complications of paediatric cancers are a substantial problem. Complications can be primary from central nervous system (CNS) spread or secondary from indirect or remote effects of cancer, as well as cancer treatments such as chemotherapy and radiation therapy. In this review, we present the clinical and imaging findings of rare but important neurological complications in paediatric patients with cancer. Neurological complications are classified into three phases: pre-treatment, treatment and post-remission. Paraneoplastic neurological syndromes, hyperviscosity syndrome, haemophagocytic lymphohistiocytosis and infection are found in the pre-treatment phase, while Trousseau’s syndrome, posterior reversible encephalopathy syndrome and methotrexate neurotoxicity are found in the treatment phase; though some complications overlap between the pre-treatment and treatment phases. Hippocampal sclerosis, radiation induced tumour, radiation induced focal haemosiderin deposition and radiation-induced white matter injury are found in the post-remission phase. With increasingly long survival after treatment, CNS complications have become more common. It is critical for radiologists to recognise neurological complications related to paediatric cancer or treatment. Magnetic resonance imaging (MRI) plays a significant role in the recognition and proper management of the neurological complications of paediatric cancer.

**Teaching Points:**

• *Neurological complications of paediatric cancer include various entities.*

• *Neurological complications are classified into three phases: pre-treatment, treatment and post-remission.*

• *Radiologists should be familiar with clinical and imaging findings of neurological complications.*

• *MRI features may be characteristic and lead to early diagnosis and proper treatments.*

## Introduction

Malignant tumours are the most common disease-related cause of death for the people under 20 years of age. With survival rates rapidly increasing with new treatments, currently two-thirds of children who suffer from paediatric cancers become long-term survivors. As survival rates for children with cancer have improved, so have the number of people who develop complications, with at least 70% of paediatric cancer survivors having some complication within 30 years from the onset their disease [1] (Table 1). Leukaemia and central nervous system (CNS) tumours make up the majority of paediatric malignancies. The most common types of brain tumours of children are astrocytoma, medulloblastoma and ependymoma [2]. Because current treatments for brain tumours and leukaemia include intrathecal chemotherapy and cranial irradiation, which are potentially neurotoxic, neurological complications are often found in patients with paediatric cancer [3]. It is necessary to be familiar with the complications that can be seen on imaging related to cancers and their treatment, as imaging is essential for early diagnosis and proper treatment to minimise adverse effects. In this review, we present the clinical and imaging findings of rare but important neurological complications in paediatric patients with cancer in the pre-treatment, treatment and post-remission phases.

**Table 1:** Neurological complications associated with paediatric cancer

Pre-treatment phase	Paraneoplastic neurological syndromes^a^
	Hyperviscosity syndrome
	Haemophagocytic lymphohistiocytosis^a^
	Infection^a^
Treatment phase	Trousseau’s syndrome^b^
	Posterior reversible encephalopathy syndrome
	Methotrexate neurotoxicity
Post-remission phase	Hippocampal sclerosis
	Radiation induced tumour
	Radiation induced focal haemosiderin deposition
	Radiation induced white matter injury

^a^Sometimes seen in treatment phase

^b^Sometimes seen in pre-treatment phase

## Pre-treatment phase

### Paraneoplastic neurological syndromes

Paraneoplastic neurological syndromes are defined as neurological syndromes caused by an autoimmune mechanism in cancer patients. Diagnosis of a paraneoplastic syndrome in pre-treatment patients must exclude local neuropathy due to metastasis, opportunistic infections accompanying decreased immune response, vascular disorders with coagulopathy and neuropathy accompanied by malnutrition. It should be noted that paraneoplastic syndromes are also sometimes found in the treatment phase. Although paraneoplastic syndromes can affect any part of the neuraxis in children, the CNS is the most commonly affected [4].

The most common paraneoplastic syndromes in children are: (1) opsoclonus myoclonus syndrome (OMS), (2) limbic encephalitis, and (3) anti-*N*-methyl-D-aspartate (NMDA) receptor encephalitis [5]. Regarding OMS, neuroblastoma is found in as many as 50% of children with the disease, although OMS occurs in just 2-3% of children with neuroblastoma [4]. The most frequently associated neoplasms with limbic encephalitis in children are neuroblastoma, Hodgkin lymphoma, ovarian teratoma and testicular tumour. Manifestation of paraneoplastic limbic encephalitis precedes the detection of cancer in 60% of patients [4]. Anti-NMDA receptor encephalitis is a paraneoplastic syndrome associated with teratomas. Most patients with anti-NMDA receptor encephalitis have full or substantial recovery after treatment. Although the most frequently associated neoplasm is ovarian teratoma, others such as mediastinal teratomas, testicular tumours and small cell lung cancers have been reported.

Typical features of limbic encephalitis include personality changes, irritability, seizures, cognitive dysfunction and memory deterioration. Children with anti-NMDA receptor encephalitis have behavioural or personality changes, sleep dysfunction, dyskinesia, dystonia and/or dysautonomia.

**Fig. 1:**
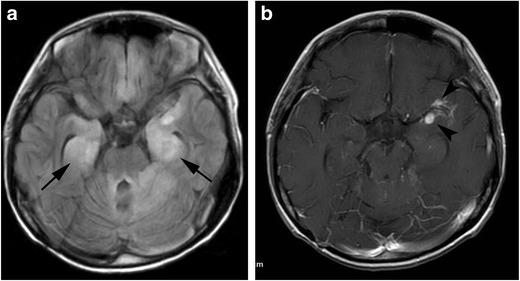
Paraneoplastic limbic encephalitis. A 17-year-old boy with recurrent medulloblastoma presented with seizure and altered mental status. **a** FLAIR image shows hyperintensities in the mesial temporal regions (*arrows*). **b** Contrast enhanced fat-suppressed T1-weighted image shows nodular meningeal enhancements in the left Sylvian fissure (*arrowheads*), suggestive of recurrence

Magnetic resonance imaging (MRI) demonstrates high signal intensity on T2-weighted images in one or both mesial temporal lobes. MRI may have abnormal signal intensities in other regions, such as the brainstem, hypothalamus, thalamus and cingulate gyrus (Fig. 1) [6]. The diagnosis of paraneoplastic limbic encephalitis must be made comprehensively in combination with the imaging and clinical findings, including cerebrospinal fluid examination, because it is difficult to distinguish from other diseases such as herpes simplex encephalitis or convulsive encephalopathy which have similar imaging features. In anti-NMDA receptor-related encephalopathy, MRI may demonstrate hyperintensities in the medial temporal lobes, cortical and subcortical regions, basal ganglia, brain stem and cerebellum on fluid attenuated inversion recovery (FLAIR) images. These regions may also show contrast enhancement [7].

Treatment is usually with immunomodulators such as steroids or intravenous immunoglobulins and plasmapheresis, along with treatment of the primary tumour.

### Hyperviscosity syndrome (HVS)

HVS has been recognised in patients with macroglobulinaemia, a type of non-Hodgkin lymphoma, since it was first described by Waldenstrom in 1944 [8]. When the serum viscosity rises, blood flow is delayed and peripheral circulation disturbance occurs. The classic triad for HVS consists of bleeding, neurological symptoms and visual disturbances. However, in addition to these symptoms, a variety of end organ damage can be observed.

Primary macroglobulinaemia is the most common cause of HVS, which accounts for 85-90% of HVS. Multiple myeloma is the second leading cause overall. While multiple myeloma can occur in children, it is exceedingly rare. HVS can also be caused by leukaemia because hyperleukocytosis, which can lead to leukostasis, is often found in leukaemia. Hyperleukocytosis is found in 5-13% patients with acute myeloid leukaemia and 10-30% patients with acute lymphocytic leukaemia [9].

**Fig. 2:**
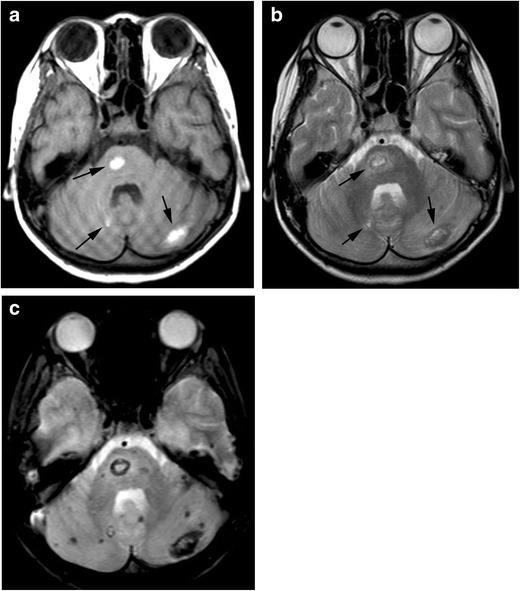
Hyperviscosity syndrome. A 10-year-old girl with acute lymphocytic leukaemia, whose white blood cell count reached 400,000, presented with headache and vomiting. **a** A T1-weighted image shows hyperintensity in the pons and bilateral cerebellar hemispheres (*arrows*). **b** These lesions show hyperintensity with a hypointensity rim on T2-weighted image (*arrows*), indicating haemorrhage in the subacute phase. **c** T2*-weighted image demonstrates a number of microhaemorrhages which are not seen on T1- and T2-weighted images

In patients with HVS, congestive heart failure, renal dysfunction, anorexia, fatigue and weakness may occur in addition to the classic triad. The most frequent symptom is bleeding, especially gingival and nasal. Although the number of platelets is usually near-normal, repetitive bleeding is characteristic. Bleeding may result from blood vessel wall abnormality caused by an intravascular friction phenomenon due to the increased blood viscosity. Neurological symptoms are common in hyperviscosity syndrome, including headache, dizziness, vision disorder, gait disturbance, sensory deafness, convulsions, coma and cerebral infarction. MRI may show multifocal parenchymal microhaemorrhages or frank haematoma formation [10, 11] (Fig. 2).

Plasma exchange therapy is widely used for the treatment of hyperviscosity syndrome, and clinical symptoms improve by decreasing blood viscosity [12]. In primary macroglobulinaemia and multiple myeloma, it is necessary to use chemotherapy in addition to plasma exchange.

### Haemophagocytic lymphohistiocytosis (HLH)

HLH is a disease characterised by systemic proliferation of histiocytes. HLH is divided into the familial/primary and the secondary types.

In both primary and secondary HLH, cytokinaemia associated with excessive activation of NK cells and cytotoxic T cells and accompanying tissue damage is the hallmark of the disease. When severe, infiltration into the central nervous system occurs. Histopathological findings of CNS invasion of HLH are classified into stages I-III. Stage I primarily shows only leptomeningeal infiltrates of lymphocytes and histiocytes/macrophages. Stage II shows additional parenchymal involvement with perivascular infiltrations. Stage III reveals cerebral tissue necrosis and demyelination in addition to massive white matter infiltration [13]. Familial HLH is genetic, mainly due to abnormality of perforin, MUNC13-4, syntaxin or MUNC18-2 [14]. Secondary types are associated with malignancy, infection, autoimmune disease and drugs. Although the most common cause of malignancy associated HLH is lymphoma and leukaemia, malignant solid tumours also cause HLH [15, 16]. Malignancy associated HLH is usually seen in the pre-treatment phase; however, it may also be seen in the treatment phase.

HLH is associated with fever, hepatosplenomegaly, lymphadenopathy, rash and bleeding tendency. Laboratory and pathological examinations reveal blood cell phagocytosis of bone marrow, pancytopenia, liver dysfunction, hypertriglyceridaemia, low fibrinogen plasma and high value of serum ferritin.

**Fig. 3:**
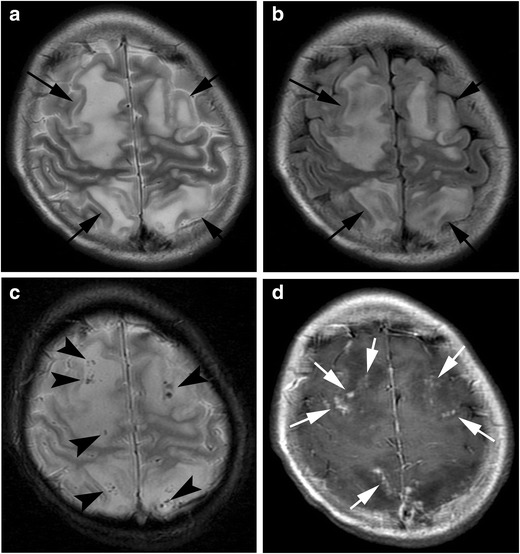
Haemophagocytic lymphohistiocytosis. A 14-year-old girl with myelodysplastic syndrome presented with seizure. **a** A T2-weighted image shows patchy hyperintensities with swelling in the frontal and parietal lobes (*arrows*). **b** FLAIR image shows hyperintensity of these lesions (*arrows*). **c** T2*-weighted image shows a number of microhaemorrhages in these lesions (*arrowheads*). **d** Post-contrast image shows nodular enhancement along the leptmeninx (*white arrows*)

MRI include diffuse leptomeningeal and perivascular enhancement, which corresponds to meningeal and perivascular infiltrations of histiocytes and lymphocytes, patchy areas of an increased T2 signal intensity in the white matter of the both cerebral hemispheres, and diffuse cerebral and cerebellar parenchymal volume loss (Fig. 3). In some cases, nodular or ring enhancement of parenchymal lesions occurs due to the compromised blood-brain barrier in areas of active demyelination. Diffusion-weighted imaging (DWI) shows diffusion restriction in white matter lesions during the acute phase [17]. Differentiation from posterior reversible encephalopathy syndrome (PRES) is often a problem both clinically and radiologically.

Although treatments of HLH have not been established, initial goals of treatments in HLH have been to suppress the overactive immune system, thus preventing immune-mediated organ damage. Patients with malignancy associated HLH require control of the HLH followed by treatment of the underlying malignancy.

### Infection

Control of infection is central in the management of cancer. Even before treatment, paediatric patients with malignant tumours are prone to infection because of decreased granulocytes, mucositis and reduced mucociliary clearance or depletion of physiological flora, together with immunosuppression due to primary disease.

Intracranial infection may result from direct spread from sinusitis or from haematogenous spread, especially in the form of septic emboli complicating endocarditis. Such infections may be fungal, bacterial or viral.

#### Fungal infection

Fungal infection typically affects children having absolute granulocyte counts of less than 100/mm^3^ for longer than 2 weeks [18].

The MRI appearance varies with the causative agent. *Aspergillus* may cause an infectious vasculopathy, leading initially to acute multiple infarctions or haemorrhage and later to extension into surrounding tissue as an infectious cerebritis or occasionally evolving into an abscess [19]. Fungal abscesses may have central restricted diffusion because of proteinaceous fluid and cellular infiltration in the lesions [20]. Haemorrhage is found in 25% of patients [21]. The contrast enhancement of the lesion is strong in immunocompetent patients but is often characteristically weak in immunocompromised patients [19]. Typical sites of involvement in *Aspergillus* vasculopathy include the basal ganglia, thalami and corpus callosum, reflecting a predisposition to involve the perforating arteries, as well as the subcortical regions. Encasement of intracranial arteries and vasculitis is found on MR angiography. Candidiasis may cause numerous microabcesses at the grey-white matter junction, basal nuclei and cerebellum, while haemorrhage and infarction are relatively rare [22]. Formation of numerous abscesses can be seen in nocardiosis, resulting in hydrocephalus, epistaxis and meningitis.

Because fungal culture tests are time-consuming and often do not lead to definitive diagnosis, empirical antifungal treatments are recommended for high-risk patients.

#### Bacterial infection

Although bacterial infections of the CNS are less common than fungal disease in immunocompromised patients with cancer, infection with *Listeria monocytogenes* and *Bacillus cereus* is well known [23, 24]. Clinical symptoms, cerebral spinal fluid (CSF) examination and laboratory data are important for the diagnosis of bacterial meningitis.

**Fig. 4:**
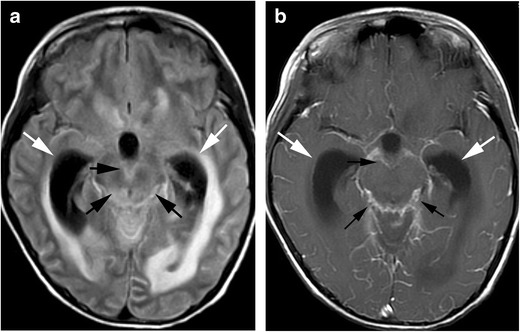
Pyogenic meningitis. An 8-year-old boy with acute myeloid leukaemia, who had received chemotherapy including intensification treatment, presented with esotropia. **a** FLAIR image shows hyperintensities along the surface of the brain stem (*arrows*). Communicating hydrocephalus is also seen (*white arrows*). **b** These lesions show enhancement (*arrows*)

MRI is useful for detecting cerebral oedema, subdural effusions/abscess and arterial or venous infarction associated with meningitis. On MRI, hyperintensities are shown in the subarachnoid spaces on the FLAIR imaging, reflecting an increase in protein concentrations. Meningeal enhancement is observed on contrast MRI (Fig. 4), and it may progress to brain abscess if meningeal inflammation spreads to the brain parenchyma. In brain abscess, sudden onset headache and focal nervous disturbance like motor paralysis, convulsions, visual disturbance and cerebellar ataxia are observed. Progression of clinical symptoms in a matter of hours is characteristic for brain abscess. Fever is recognised only in about half of cases. MRI shows iso- or hyper-intensity on T1-weighted images with a low signal intensity rim on T2-weighted images. Post-contrast images show ring enhancement. Marked central diffusion restriction and rapid growth can help to differentiated abscess from neoplasm.

Treatment with broad-spectrum antibiotics with narrowing of coverage as possible is the general approach to treatment.

#### Viral infection

The most common pathogen in viral infection in patients with haematological malignancy is the herpes virus. Among the herpes subtypes, HHV-6 is important as an opportunistic infection in immunosuppressed patients [25]. HHV-6 infection is present in 90% of children by 2 years of age. While inactive at sanctuary sites in the parotid gland and brain, HHV-6 activation leading to encephalitis occurs in immunosuppressed patients.

MRI findings of HHV-6 encephalitis are similar to acute disseminated encephalomyelitis, and scattered hyperintensities are seen in cerebral white matter on T2 weighted images [26].

Treatment is using with anti-viral agents such as acyclovir and gancyclovir, which are often started empirically when the imaging and clinical findings are suggestive.

## Treatment phase

### Trousseau’s syndrome (cancer-associated thrombosis)

Trousseau’s syndrome was first reported in 1865 by Armand Trousseau as a condition of cerebral infarction and pulmonary embolism due to multiple venous thrombosis associated with gastric cancer. In 1977, Sack et al. reported that Trousseau’s syndrome is chronic disseminated intravascular coagulation (DIC) associated with non-bacterial thrombotic endocarditis and arterial thrombosis in patients with malignancy. Currently, the term “Trousseau’s syndrome” is often used to describe a hypercoagulation disorder associated with various malignancies. It is reported that the risk of venous thromboembolism, including both deep vein thrombosis and pulmonary embolism, is fourfold to sevenfold higher in patients with cancer than those without cancer [27]. Neurological symptoms depend on the infarcted area; however, altered mental status and convulsion are often observed. Although Trousseau’s syndrome is classified as a treatment phase complication in this article, it can occur as a primary symptom in some patients with paediatric cancer.

The brain is abundant with thromboplastin, which triggers the exogenous coagulation cascade and is thought to be a target of DIC due to the lack of thrombomodulin, a thrombin antagonist. Although the mechanism of hypercoagulability in patients with cancer has not been fully elucidated, it is thought that tumour cells express tissue factors that activate the coagulation cascade, including cellular procoagulants such as tumour procoagulant and factor X receptors. These lead to thrombosis by inducing cell-cell interactions with platelets, monocytes and endothelium via inflammatory cytokines, tumour antigens, and their immunoconjugates, which promote coagulation activation. Although malignant tumours that cause Trousseau’s syndrome tend to be solid, children with any malignancy are at increased risk [28].

**Fig. 5:**
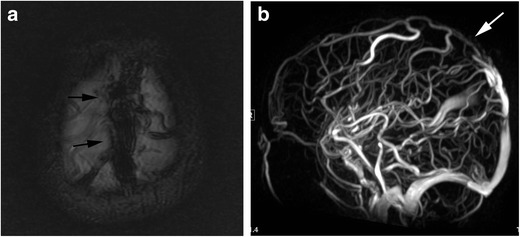
Trousseau’s syndrome. (dural sinus thrombosis). A 5-year-old boy with acute lymphocytic leukaemia presented with seizure and altered mental status. **a** A T2*-weighted image shows hypointensity in the superior sagittal sinus (*arrows*). **b** MR venography shows signal loss in the superior sagittal sinus (*white arrow*)

Certain MRI features can suggest this entity. Trousseau’s syndrome should be considered, particularly when cerebral infarction involves three or more vascular territories. Microemboli are usually scattered in multiple vascular territories [29, 30]. In addition, MRI with MR venography is useful for the diagnosis of dural sinus thrombosis. MR venography can show loss of the flow void in an affected dural sinus [31] (Fig. 5). Since there are no clear diagnostic criteria for Trousseau’s syndrome, it is important to search for malignant tumours when unexplained cerebral infarction or sinus thrombosis are found.

Regarding therapy, in addition to treating the underlying cancer, anticoagulant therapy using heparin is also usually necessary; warfarin is usually ineffective.

### Posterior reversible encephalopathy syndrome (PRES)

PRES is an acute neurotoxic syndrome of characteristically reversible subcortical vasogenic brain oedema in patients with acute neurological symptoms. The pathophysiology of PRES is thought to be due to failure of cerebral blood flow autoregulation from endothelial dysfunction [32]. Predisposing conditions associated with PRES in patients with cancer include chemotherapeutics or immunosuppressant administration, infection and autoimmune disorders. Hypertension is associated with paediatric PRES, but because the cerebral blood flow autoregulation threshold is lower in children than in adults, the mean blood pressure at the onset of PRES is also lower [33]. The mean blood pressure at the onset of paediatric PRES was reported to be 140/85 mmHg [34]. The spectrum of neurological features observed in patients with PRES includes headache, seizure, visual disturbances and nausea.

**Fig. 6:**
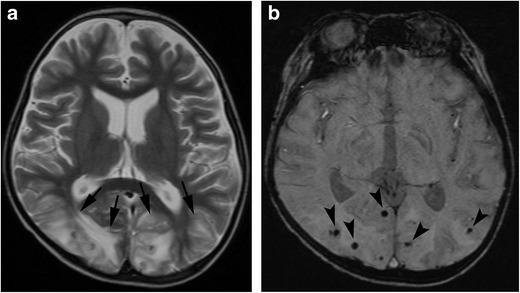
Posterior reversible encephalopathy syndrome. (PRES). A 5-year-old boy with acute lymphocytic leukaemia, who received bone marrow transplantation, developed lethargy during administration of tacrolimus. **a** A T2-weighted image shows hyperintensities in the occiptal lobes (*arrows*). **b** A susceptibility-weighted image (SWI) shows punctate signal loss in these lesions, suggestive of microhaemorrhage (*arrowheads*)

Although the subcortical white matter and cortex are often involved, distribution of abnormal imaging findings in PRES is classified into four patterns: holohemispheric watershed, superior frontal sulcus, dominant parietal-occipital and partial or asymmetric [35]. The basal ganglia, brain stem and cerebellum are also sometimes involved. MRI shows regions of high signal intensity on T2-weighted or FLAIR images. Restricted diffusion can be seen in 15-30% of cases, which is generally associated with irreversible change [32] (Fig. 6). Contrast enhancement is seen in about 20% of patients with PRES. Intraparechymal or subarachnoid haemorrhage around cortical or subcortical lesions is seen in 10-25% of cases. Intraparenchymal haemorrhage is often multifocal; however, mass effect is rare. Subarachnoid haemorrhage spares the basilar cisterns [32, 36]. Additionally, temporal lobe involvement, restricted diffusion on MRI, and associated multi-organ failure are more frequent in paediatric PRES compared with adults [37].

Treatment of PRES depends on its cause. Anti-epileptic medication may also be appropriate.

### Methotrexate neurotoxicity

Abnormalities of the cerebral white matter are seen in some patients following treatment with chemotherapeutic agents. Although several drugs cause leukoencephalopathy, methotrexate (MTX) is the most common one in children. MTX is an antifolate drug used for treatment of diseases such as acute lymphoblastic leukaemia, malignant lymphoma and sarcoma. MTX neurotoxicity is found in 3-10% of recipients.

It is believed that MTX can induce direct toxic effects to the CNS by damaging the neuronal tissue. Moreover, MTX interferes with the metabolic pathways of folate and induces biochemical alterations in excitatory amino acids, homocysteine, S-adenosylmethionine/S-adenosylhomocysteine, adenosine and biopterins [38], which can lead to neurological symptoms. High-dose intravenous administration, intrathecal administration, teenage and a history of radiation therapy are risk factors.

Its neurotoxicity can be classified as acute, subacute and chronic. Acute or subacute neurotoxicity can present with stroke-like symptoms such as aphasia, muscle weakness, sensory disturbance and ataxia, occurring within 2-14 days after initiation of MTX. Neurological symptoms are usually transient. In contrast, the chronic type can cause a slowly developing leukoencephalopathy and may progress to permanent impairment of neurological function.

**Fig. 7:**
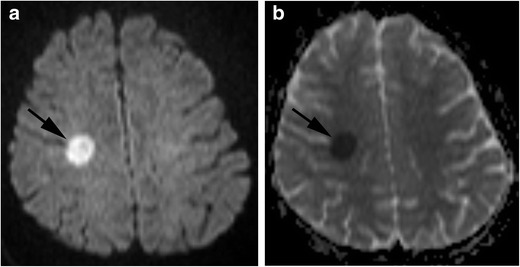
Methotrexate neurotoxicity. (acute phase). A 14-year-old girl with acute lymphocytic leukaemia, who received intrathecal methotrexate, presented with left hemiplegia. **a** Diffusion-weighted image shows a small area of hyperintensity within the right centrum semiovale (*arrow*). **b** Apparent diffusion coefficient map shows restricted diffusion in this lesion (*arrow*)

MRI of acute neurotoxicity shows diffusion restriction on DWI in cerebral white matter, especially in the centrum semiovale or corona radiata, indicating intramyelinic oedema (Fig. 7). Although T2-weighted and FLAIR images show hyperintensities, they may be quite subtle. DWI findings are normal after recovery, while T2 and FLAIR images usually show slight residual abnormalities. The cerebral cortex and cerebellum are also involved in atypical cases [39]. When imaging findings resembling cerebral infarction appear in patients receiving MTX, MTX neurotoxicity should be suspected.

After cessation of MTX and resolution of the associated neurotoxicity, subsequent intrathecal MTX administration is not associated with recurrence of MTX neurotoxicity.

## Post-remission phase

### Hippocampal sclerosis

Hippocampal sclerosis is a neuropathological condition with severe neuronal cell loss and gliosis in the hippocampus, specifically in the cornu ammonias area 1 and subiculum of the hippocampus. Although hippocampal sclerosis is generally associated with biphasic seizures of the infantile period, cerebritis, head trauma and perinatal brain injury, there are also some reports of hippocampal sclerosis in patients with haematological malignancy [40]. Various factors are thought to underlie the sclerosis seen in haematological malignancies, such as neurotoxicity due to methotrexate or cyclosporine, radiation exposure and bone marrow transplantation. Hippocampal sclerosis accounts for a large portion of refractory temporal lobe epilepsy in children. Although hippocampal sclerosis is highly associated with mesial temporal lobe epilepsy, it is not known whether hippocampal sclerosis causes temporal lobe epilepsy or temporal lobe epilepsy causes hippocampal sclerosis.

**Fig. 8:**
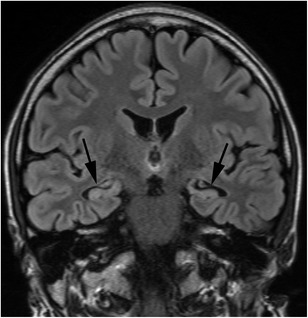
Hippocampal sclerosis. A 24-year-old-man, who received bone marrow transplantation for malignant lymphoma at the age of six, presented with temporal lobe epilepsy. FLAIR image shows atrophy with high signal intensity in the bilateral amygdalae and hippocampi (*arrows*)

On MRI, coronal FLAIR and short tau inversion recovery images perpendicular to the hippocampus are useful. Atrophy and high signal intensity of the hippocampus and amygdala are observed (Fig. 8). In temporal lobe epilepsy, slight abnormal signal may be observed in the white matter at the temporal lobe tip, leading to the diagnosis of hippocampal sclerosis in the initial stages [41].

Medial temporal lobe epilepsy is usually refractory to anticonvulsant drugs; brain surgery is effective in such patients. Hence, early detection of hippocampal sclerosis in patients with haematological cancer is important, because these children are likely candidates for brain surgery.

### Radiation induced tumours

Development of a secondary neoplasm is an important late complication of radiation therapy. Childhood cancer survivors who received cranial radiation therapy have an 8.1–52.3 times higher incidence of subsequent CNS neoplasms compared with the general population [42]. In adult patients with radiation induced brain tumours, meningiomas represent approximately 70%, gliomas 20% and sarcomas 10% [43]. In paediatric populations, high-grade gliomas and meningiomas are the two most common subsequent CNS neoplasms, although medulloblastomas, primitive neuroectodermal tumours (PNETs), schwannomas and low-grade gliomas have also been reported [42]. Initial studies from the early 1990s had suggested that high-grade gliomas occur in the 1st decade after primary cancer diagnosis, but more recent studies with longer follow-up have shown that high-grade gliomas also occur in the 2nd decade after primary cancer therapy. Moreover, survivors of brain tumours who had not developed meningiomas at 20 years after diagnosis of their original cancer still had a 5.3% incidence of meningiomas in the subsequent decade [42]. Thus, deciding on an optimal screening regimen is challenging.

**Fig. 9:**
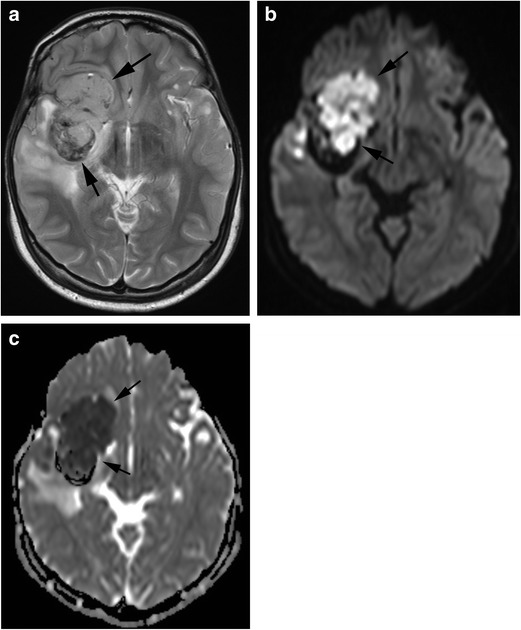
Radiation induced atypical teratoid/rhabdoid tumour. A 21-year-old man, who received radiation therapy for optic glioma during infancy, presented with headache, appetite loss and somnolence. **a** A T2-weighted image shows a lobulated, solid mass with internal low signal intensity, indicating a small haemorrhage in the middle cranial fossa to basal ganglia (*arrows*). **b** Diffusion weighted image shows hyperintensity in the tumour (*arrows*). **c** Apparent diffusion coefficient map shows restricted diffusion in the tumour (*arrows*)

There is a recommendation that secondary brain tumour screening by MRI should be performed annually for the initial 5 years after completion of the radiotherapy, and thereafter repeated MRI screening should be performed only for the patients with neurological symptoms, such as headache, cognitive changes and seizures, particularly in those with a history of haematological malignancy or radiotherapy at a young age [44]. However, large sample, prospective randomised studies are needed in this regard. Radiation-induced gliomas are associated with high-grade gliomas in young people, multiplicity of gliomas and earlier age at presentation [45]. The neuroimaging appearance of radiation-induced tumours does not differ from that of other types (Fig. 9).

### Radiation induced focal haemosiderin deposition (RIFHD)

RIFHD is a late complication of radiation therapy, which represents haemorrhagic or proliferative microangiopathies such as capillary telangiectasias and cavernous malformations [46]. Pathologically, telangiectasias and cavernous malformations differ only in the presence or absence of intervening brain parenchyma among the dilated, thin-walled vascular channels. Because these similarities and transitional forms of these vascular malformations have been observed in some patients, radiation-induced telangiectasias and cavernous malformations have been proposed to exist along a spectrum driven by a common proliferative pathway [47].

The mechanism of RIFHD probably involves vascular injury, proliferation and dilation of vascular endothelium, hyalinisation and fibrinoid necrosis of blood vessel walls due to radiation therapy, and finally ischaemia and infarction due to narrowing of the vascular lumen [48]. The influence of age at the time of radiation therapy varies by report, and there is no consensus [46, 49, 50]. Radiation dose of 6-12 Gy has been reported as the minimum threshold level for development of RIFHD, and radiation dose positively correlates with frequency of RIFHD [50]. Although most patients with RIFHD are asymptomatic, symptomatic bleeding may occur. Infratentorial RIFHD is more prone to symptomatic bleeding compared with supratentorial RIFHD [51, 52]. RIFHD is associated with neurocognitive dysfunction in primary brain tumour survivors [53].

**Fig. 10:**
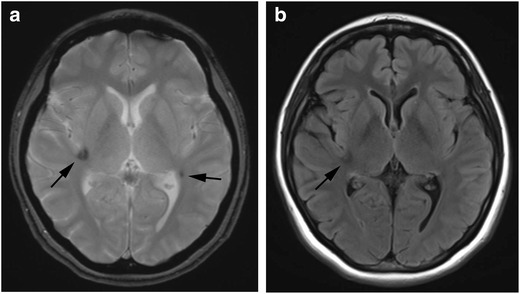
Radiation induced focal haemosiderin deposition. A 29-year-old woman, who received radiation therapy for acute lymphocytic leukaemia, had no symptom. **a** A T2*-weighted image shows focal haemosiderin deposition in the right insular and left temporo-parietal lobe (*arrows*). **b** FLAIR image shows slight low intensity area in the right insular (*arrowheads*). Left temporo-parietal small lesion is not apparent (*arrow*)

On MRI, RIFHD shows mixed intensity with an enhancing cystic and/or solid component and an incomplete haemosiderin rim, which would be insufficient for a diagnosis of de novo cavernous malformation [54]. Small RIFHD lesions are best seen on iron-sensitive sequences such as gradient recalled echo imaging or susceptibility weighted imaging [48] (Fig. 10).

The treatment algorithm for RIFHD is not well established because the natural course of RIFHD has yet to be elucidated. Surgery may be considered in patients with repeated bleeding episodes and progressive neurological symptoms.

### Radiation induced white matter injury

White matter injury is also an important late complication of radiotherapy. Radiation-induced white matter injury is divided into acute, early-delayed and late-delayed injury [55]. Acute and early-delayed reactions are often mild. Late-delayed injury is found as early as 3-4 months or as late as several years after completion of therapy.

It is believed to result mainly from permanent damage to blood vessels. Patients may develop progressive neurological symptoms. Pathologically, the affected white matter exhibits necrosis, with rarefaction and fragmentation of myelin and cellular disruption.

**Fig. 11:**
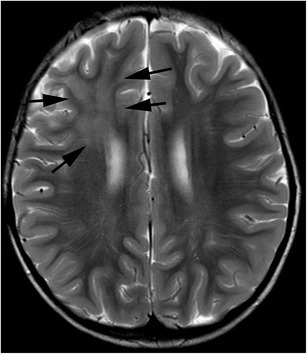
Radiation induced white matter injury. A 5-year-old boy, who received radiation therapy for anaplastic ependymoma, was asymptomatic. A T2-weighted image shows hyperintensity in the right frontal lobe which was irradiated (*arrows*)

Imaging studies show variable patterns of injury, such as focal lesions or diffuse white matter abnormality. MRI demonstrates hypointensity on T1-weighted images and hyperintensity on T2-weighted images (Fig. 11). In whole-brain radiation, signal changes occur in the periventricular region and may progress in size and signal intensity over time, extending peripherally to the subcortical fibres [56]. Telencephalic commissural fibres are typically spared [56]. White matter volume loss may occur as a result of diffuse radiation injury [56]. Central necrosis within the lesions is uncommon in children [56].

## Conclusions

Neurological complications of paediatric cancer are a substantial problem and are associated with significant morbidity and loss of quality of life for long-term survivors of paediatric cancer. It is critical for radiologists to recognise the imaging findings of these rare but important complications related to disease and treatment. MRI plays a significant role in the recognition and proper management of neurological complications of paediatric cancer.
